# Enzyme-Crosslinked Gelatin Hydrogel with Adipose-Derived Stem Cell Spheroid Facilitating Wound Repair in the Murine Burn Model

**DOI:** 10.3390/polym12122997

**Published:** 2020-12-16

**Authors:** Ting-Yu Lu, Kai-Fu Yu, Shuo-Hsiu Kuo, Nai-Chen Cheng, Er-Yuan Chuang, Jia-Shing Yu

**Affiliations:** 1 Department of Chemical Engineering, College of Engineering, National Taiwan University, Taipei 10617, Taiwan; bbb32037@gmail.com (T.-Y.L.); b04504122@gmail.com (K.-F.Y.); b05504084@ntu.edu.tw (S.-H.K.); 2 Department of Surgery, National Taiwan University Hospital, Taipei 10617, Taiwan; 3 International Ph.D. Program in Biomedical Engineering, Graduate Institute of Biomedical Materials and Tissue Engineering, Taipei Medical University, Taipei 11031, Taiwan; 4 Cell Physiology and Molecular Image Research Center, Wan Fang Hospital, Taipei Medical University, Taipei 11031, Taiwan

**Keywords:** gelatin, hydrogel, human adipose-derived stem cells, cell spheroid, wound healing

## Abstract

Engineered skin that can facilitate tissue repair has been a great advance in the field of wound healing. A well-designed dressing material together with active biological cues such as cells or growth factors can overcome the limitation of using auto-grafts from patients. Recently, many studies showed that human adipose-derived stem cells (hASCs) can be used to promote wound healing and skin tissue engineering. hASCs have already been widely applied for clinical trials. hASCs can be harvested abundantly because they can be easily isolated from fat tissue known as the stromal vascular fraction (SVF). On the other hand, increasing studies have proven that cells from spheroids can better simulate the biological microenvironment and can enhance the expression of stemness markers. However, a three-dimensional (3D) scaffold that can harbor implanted cells and can serve as a skin-repaired substitute still suffers from deficiency. In this study, we applied a gelatin/microbial transglutaminase (mTG) hydrogel to encapsulate hASC spheroids to evaluate the performance of 3D cells on skin wound healing. The results showed that the hydrogel is not toxic to the wound and that cell spheroids have significantly improved wound healing compared to cell suspension encapsulated in the hydrogel. Additionally, a hydrogel with cell spheroids was much more effective than other groups in angiogenesis since the cell spheroid has the possibility of cell–cell signaling to promote vascular generation.

## 1. Introduction

### 1.1. Gelatin as an In Situ Scaffold Material for Regeneration

In recent years, research regarding tissue engineering technology and regeneration of engineered biopolymers to three-dimensional (3D) structures has received extensive attention [1,2]. A well-designed scaffold with an appropriate porous structure can be used as a 3D specimen for initial cell adhesion and consecutive tissue formation [3]. The scaffold can also be used as an ideal skin substitute for wound regeneration that supports cell migration and leads to blood vessel infiltration [4,5,6]. Mean pore size is an essential aspect of scaffolds for tissue engineering. Many in vitro studies focused on determination of the optimal average pore sizes of scaffolds. Moderate pores can help cells migrate towards the center of the construct, limiting diffusion of nutrients and removal of waste products. Moreover, pores in the scaffold can also increase the surface area available for cell attachment. It was shown that pore sizes of biomaterial scaffolds ranging from 50–300 μm facilitated cell adhesion, proliferation, and migration [7,8].

Hydrogel is well-known for moist wound healing, with its transparency and good fluid absorption, which allow for healing monitoring. There are many hydrogel patch materials available and under study [9]. Compared with synthetic polymers, many researches showed that biopolymer scaffolds are capable of regulating cell division, adhesion, differentiation, and migration [10]. Gelatin is a natural biopolymer derived from collagen through controlled hydrolysis. It shows significant advantages in tissue engineering applications, including good biocompatibility, water holding capacity, matrix metalloproteinase (MMP)-mediated degradation, and natural retention cell adhesion motif [11,12]. Thus, various studies indicate that gelatin is a suitable topical regenerative biomaterial for wound repair, and gelatin hydrogels are widely applied for hydrogel patches for wound repair.

### 1.2. Burn Wound Care

Skin is a multi-layered organ that can act as a barrier to guard against dehydration, to protect the body from pathogens, and to perform biological functions. However, the skin is highly vulnerable to frostbite, electricity, chemicals, and heat [13]. Acute thermal injuries are the most common harm to human skin. According to the latest information from the World Health Organization, burns cause nearly 180,000 deaths a year [14]. With modern medical technology, the survival rate has been raised significantly; however, serious burns are still hard to manage and usually come with long-term hospitalization and expensive treatment options. In recent decades, promising strategies such as tissue engineering alternatives based on mesenchymal cells (Adipose Stem Cells (ASCs)) have been widely used in clinical trials or laboratories [15]. The role of ASCs and adipose tissue in the repair of complex burn wounds has been documented, including for inflammation, granulation, and remodeling [16].

### 1.3. Stem Cells as a Wound Treatment

Stem cells are the body’s raw materials which can generate other cells with specialized functions. Under appropriate conditions, the stem cell can differentiate into multiple cell types and has the ability to self-renew. Utilizing stem cells to regenerate damaged organs and tissues brings hope to the treatment of various diseases which were ineffective in the past. Applying stem cells to wound healing to promote skin regeneration, especially in burns, has also triggered researchers’ interest in the field of wound healing [17,18,19]. Mesenchymal stem cells (MSCs) are pluripotent stem cells with the ability to differentiate. MSC is the most widely used cell in the field of regenerative medicine. MSCs play an important role in tissue repair and maintenance and have been broadly studied because of their vital role in enhancing the regeneration capacity of many tissues in regenerative therapy [20,21]. A large amount of evidence from animal studies shows the redundancy of growth factors and cytokines working together in MSC-secreted growth factors, for instance, epidermal growth factor (EGF), platelet-derived growth factor (PDGF), transforming growth factor β (TGF-β), fibroblast growth factor, and vascular endothelial growth factor (VEGF). These factors can reduce scarring, neovascularization (NV), and inflammation while promoting epithelial formation after injury [22,23]. Application of these growth factors in clinical studies further confirms their roles in wound healing.

### 1.4. Adipose Stem Cells (ASCs) as a Therapeutic Agent

Although MSCs have advantages in wound healing tissue repair, the limitations associated with this technique still exist. The traditional bone marrow harvesting process has many disadvantages. First, the traditional method causes great suffering for the patient. Second, the process requires general anesthesia or spinal anesthesia. Third, it may produce only a little bit of MSCs [24]. Additionally, research also implies that, as the donor ages, the differentiation potential of MSCs significantly decreases. Therefore, the application of adipose-derived stem cells (ASCs) is considered a more possible approach for cell transplantation in regenerative medicine [25]. Human Adipose Stem Cells (hASCs) are stem cell populations with multiple differentiation potentials. Due to the development of liposuction, hASC has become a rich source of stem cells and can be harvested safely in large quantities [26]. As confirmed by in vitro and in vivo experiments and clinical studies, hASCs mainly promote wound healing through two mechanisms. First, hASCs differentiate into target cells involved in wound healing [27]. Second, hASCs differentiate into cytokine paracrine cells to promote proliferation and migration of various cell lines essential for skin wound healing [28]. hASCs could also secrete several growth factors such as PDGF, EGF, and TGF-β [29]. Particularly, these growth factors are involved in angiogenesis in wound healing.

### 1.5. 3D Cell Spheroids

In the past few years, traditional cell culture systems which incubate cells on the two-dimensional (2D) plane have been widely used for extensive elementary in vitro cell research because of simply equipped culture conditions and the convenience of operation for microscopic analysis. However, some research has proven that a lot of cell types lose their primitive functions and characteristics like differentiation, cell growth, and biological functions under a 2D culture environment [30,31]. Therefore, many researchers made efforts to work on the development of a three-dimensional (3D) cell culture system. Intercellular interaction plays an important role in the 3D culture system, and this feature is also the biggest difference from a 2D cell culture [32]. With the current emergence of the three-dimensional cell culture, cell spheroids micro-aggregates have been shown to mimic the natural physiological environment of tissues. Compared with 2D culture, the clinical relevance and biological relevance of 3D cell spheroids are higher because of their higher regeneration potential [33]. Cell spheroid formation is a 3D cell culture method that enhances the interaction between cells and replicates interaction between cells and the extracellular cell matrix (ECM) without adding additional matrix [34]. It has recently been proven that, compared with the traditional 2D culture system, the 3D culture system successfully enhances the differentiation efficiency of hASCs [35,36].

Our previous study compared the adipogenic and chondrogenic abilities of hASC spheroids with conventional 2D hASC culture and found that the adipogenic differentiation and chondrogenic differentiation markers in spheroids were significantly upregulated. The results of differentiation analysis showed that, compared with the cell suspension group, the cell spheroids had better differentiation potential, especially adipogenesis and cartilage formation [37].

**Scheme 1:**
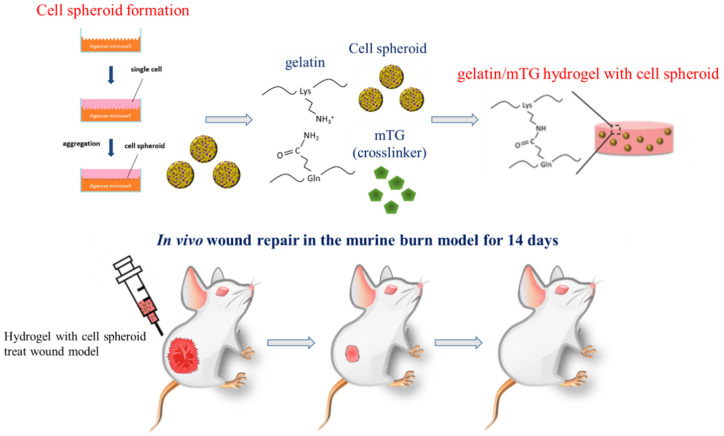
Schematic illustration of enzyme-crosslinked gelatin hydrogel with adipose-derived stem cell spheroid facilitating wound repair in the murine burn model.

In this study, we explored an in situ gelatin hydrogel system encapsulating human adipose stem cells and investigated the effectiveness of wound healing between cell spheroids and cell suspension groups. In an animal study, we evaluate the gelatin hydrogel system with hASC spheroids regarding its wound healing potential through a Wistar rat burn injury wound model. Therefore, the animal study results including the wound contraction area, histopathological examinations (Hematoxylin and Eosin (H&E)) staining, and angiogenesis effect for CD31 immunohistochemistry staining demonstrated the wound-injury recovery effects of hydrogel scaffolds with cell spheroids. All the in vivo study data prove that the regenerative biomaterials and stem cell spheroid/gelatin hydrogel system exhibit great potential as burn hydrogel patches (Scheme 1).

## 2. Materials and Methods

### 2.1. Materials

Gelatin powder (type B, Sigma, St. Louis, MO, USA), microbial transglutaminase (mTG) (from guinea pig liver, Modernist Pantry, Eliot, ME, USA), Dulbecco’s modified Eagle’s medium (DMEM) (HyClone, Logan, UT, USA), 1% penicillin/streptomycin-Amp solution (PSA; ABM, New York, NY, USA), 10% fetal bovine serum (FBS; ABM, New York, NY, USA), trypsin-EDTA (Biological Industries, Cromwell, CT, USA), basic fibroblast growth factor (bFGF; Sigma, St. Louis, MO, USA), Live/Dead kit (Thermo Fisher Scientific, Waltham, MA, USA), 10× blocking Buffer (ab126587), Anti-CD31 antibody (ab28364), Goat Anti-Rabbit IgG H&L (HRP) (ab6721), and tegaderm film (3M, Taipei, Taiwan) were used.

### 2.2. Isolation and Cell Culture of hASCs

In the liposuction operation at National Taiwan University Hospital, human subcutaneous fat tissue was obtained for cosmetic plastic surgery. The experimental protocol and surgical procedures were approved by the National Taiwan University Hospital (NTUH) Research Ethics Committee (REC). To isolate hASCs, the lipoaspirate was thoroughly washed and blood cells were lysed. First, the adipose tissue was digested using collagenase, and then, the remaining tissue was filtered and centrifuged. After that, the adipose-derived stromal vascular fraction (SVF) was collected. Under standard incubation conditions (37 °C, 5% CO_2_), the adipose-derived SVF easily adhered to the plastic tissue culture. hASCs were incubated in DMEM growth medium supplemented with basic fibroblast growth factor (bFGF) (1 ng/mL). Until cells reached 90% confluence, after PBS washing, trypsin was added and reacted at 37 °C for 5 min to separate the cells from the 150-cm^2^ cell culture flask (T150). Then, the growth medium was added to T150 to stop the enzyme reaction, and the cells were collected by centrifugation. After aspiration, the supernatant was removed and the growth medium was added to the resuspended cell pellet. A hemocytometer is used to count the number of suspended cells.

### 2.3. Formation of Cell Spheroids

To prepare the cell spheroids, first, the agarose microplate was immersed in 75% ethanol for 1 h, then immersed in PBS, and then used. Next, each agarose microplate was placed tightly in a 24-well plate, then added 1 mL of growth medium, and then centrifuged (3000 rpm) for 5 min to remove residual bubbles. Subsequently, the cell suspension was seeded with 1.0 × 10^6^ cells in each microwell plate, and the liquid was gently pipetted several times to make the cells evenly distributed throughout the microwell. After centrifuging the 24-well plate (400 rpm) for 5 min to assure that the cells were aggregated in the microwells, they were then incubated 3 days under normal condition (37 °C, 5% CO_2_) with 1 mL growth medium for cell aggregation. After accumulating the cell spheroids evenly in the agarose microplate, the suspension was gently pipetted to separate the cell spheroids and then the prepared cell spheroids were collected through a cell strainer (Bio-genesis Technologies, lnc., Taipei, Taiwan) (100 μm) [37].

### 2.4. Preparation of Gelatin/mTG Hydrogel

To prepare the gelatin/mTG hydrogel, the gelatin powder was dissolved in a growth medium under 70 °C heating and then filtered through a 0.22 μm filter. hASCs were incubated in growth medium. The cells were cultured at 37 °C, 5% CO_2_, and 99% humidity. Similarly, mTG was dissolved in the growth medium and then sterilized through a 0.22-μm filter to prepare the mTG solution. The gelatin/mTG hydrogel was prepared by mixing a 6% gelatin solution with a 60 U g^−1^ pro mTG dose.

### 2.5. Stem Cell/Hydrogel Formation

Mix solution of Gelatin (6%) and mTG (60 U g^−1^ pro) mixed with stem cells were placed into 6-well culture plates with cell density 1 × 10^6^ cells and 1 mL growth medium. After cross-linking, growth medium (3 mL) was added in every well and incubated at 37 °C, 5% CO_2_.

### 2.6. Cell Viability

After the cells were cultured for 7 days, the Live/Dead kit was used to determine the cell viability in the 3D hydrogel. The sample incubated for 1 h in a solution containing live/dead assay (2 mM calcein AM, 4 mM ethidium homodimer-1). After incubation, the hydrogel samples were observed using a confocal laser scanning microscope (Leica TCS SP5, Heidelberg, Germany).

### 2.7. Animals and Burn Wound Model

Male Wistar rat, aged 8 weeks (300–400 g), were purchased from BioLASCO Taiwan (Taipei). Animal care followed the Guidance for Care and Use of Laboratory Animals (approved number: LAC-2019-0325), approved by the Taipei Medical University Administrative Committee on Animal Research. All experiment processes were carried out under anesthetic gases (isoflurane, 2–4%). After the experiment, the animals were sacrificed by overdose of CO_2_ euthanasia. Under the inhalant anesthesia, the hair of the animal was removed through electric clippers and then their back was shaved with hair removal cream. A stainless-steel cylinder (1.0 cm in diameter) was heated to 100 °C in a water bath for 8 min and placed on the back of rats for 15 s to cause partial-thickness burn wound, and the wound region was recorded instantly. During the wound healing experiment, rats were randomly divided into five equally studied groups: group I (only hydrogel); group II and group III (hydrogel with cell suspension and hydrogel with cell spheroid), which were used to compare the effects of the hydrogel and different cell shapes on wound healing; group IV (only cell suspension); and group V (control), which is defined as the group that did not receive treatment. After treatment, the hydrogel was fixed with a tegaderm film to avoid dropping the hydrogel.

The wound-injury rats were randomly divided into the five groups (n = 5). Wound areas were recorded by digital camera on days 0, 3, 7, 10 and 14, and wound contraction rates (%) were measured by ImagePro 10 software. Wound photos were taken to measure the wound healing index and wound contraction rate. Each group was calculated by 5 blinded individuals on a score from 0–4 based on (1) brown discoloration and (2) scabbing/hardness (Table 1). The wound contraction rate was calculated through ImageJ software from pixel count of the wound area. The wound size at day 0 was defined as 100%.

### 2.8. Histological Analysis and Immunohistochemically

Wound samples were harvested after mice were sacrificed, fixed, and embedded in paraffin. Tissue sections (5 µm) were mounted on slides for histological analysis. In order to visualize the pathological changes, formed tissue, and collagen formation at different healing times, Hematoxylin and Eosin (H&E) and Masson’s trichrome staining were used. The stained slides were assessed using a fluorescence microscopy (Olympus ix83). For immunohistochemically staining, sections were deparaffinized, rehydrated, and treated with H_2_O_2_ and carbinol to block endogenous peroxidase activity. Samples were heated in a microwave oven twice to recover the antigen and were treated with blocking buffer (5% Bovine Serum Albumin (BSA) solution in Phosphate Buffered Saline with Tween^®^ 20 (PBST) to block the nonspecific reactions. Then, sections were applied in the primary antibody Anti-CD31 antibody (1:50) diluted in blocking buffer and incubated overnight at 4 °C, treated for 1 h with secondary antibody HRP goat anti-rabbit (1:200), and visualized by a DAB kit.

### 2.9. Statistics

All data are presented as mean ± SD (at least in three independent batches). Statistical significance was evaluated by Student’s *t*-test. Statistical differences between samples were performed with significance at *p* < 0.05.

## 3. Result and Discussion

### 3.1. Cell Viability and Morphology in Hydrogel

**Figure 1:**
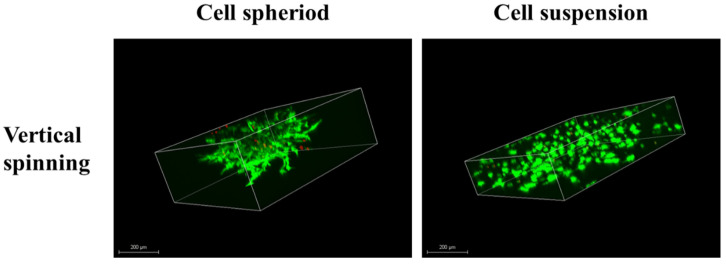
Three-dimensional live/dead staining image of human adipose-derived stem cells (hASCs) in the 3D hydrogel system after 7 day incubation (green: live and red: dead), scale bar = 200 mm.

Biocompatibility is an important indicator to evaluate whether the material will affect the organism. Therefore, the cell viability and distribution of stem cells in the 3D hydrogel were evaluated through live/dead assays that showed the viability of cells in the hydrogel and the distribution of surviving spheroid cells (Figure 1 and Figure S1 (Supplementary Materials)). The images show that the cell suspensions and cell spheroids detected fewer red signal (dead cells) and higher green signal (live cells) after 7 days of culture. We found that the encapsulated cells and cell spheroids presented good viability and that few dead cell signals were detected after 7 days of culture. For the cell suspension group, 3D confocal laser scanning microscope images showed that the cells were evenly distributed in the 3D hydrogel. By comparison, the 3D images of the cell spheroid group showed that the shape of the spheroid was no longer complete and that the cells migrate widely from the spheroid to the 3D hydrogel. In order to observe the cell viability of the hASCs grown in the 3D hydrogel system, the cell morphology images were taken by microscopy. The cell suspension group showed that hASCs spread widely after 7 days of incubation and that cell morphology had not changed significantly. For the cell spheroid group, hASCs started to migrate from the cell spheroid and proliferated after a 3-day culture. In addition, the cells also spread widely in the 3D hydrogel after a 7 day culture (Figure S2). As a result, live/dead staining demonstrated that a gelatin enzyme cross-linked hydrogel has good biocompatibility with the hASCs. Moreover, according to 3D images, cell spheroid and cell suspension groups both demonstrated that hASCs can evenly distribute and have great proliferation.

### 3.2. Evaluation of the Wound Healing Ability

**Figure 2:**
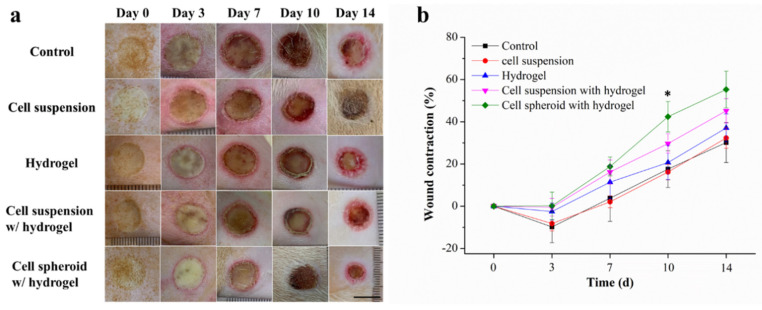
(**a**) Photographs of representative wounds and (**b**) wound contraction rates as a function of wound healing effect as estimated by ImageJ analysis of wound size (n = 4): samples were analyzed with one-way ANOVA, * *p* < 0.05. Error bars represent mean ± s.d.

Before performing histological analysis on the skin of the burn injury site, the wound areas were visualized as images and monitored at days 0, 3, 7, 10 and 14 (Figure 2a). Then, the wound contraction rate was calculated to evaluate the wound healing effect in the experimental group (Figure 2b). The cell spheroid with hydrogel group achieved the highest wound contraction rate of 55.3%, followed by cell suspension with hydrogel (45.2%), hydrogel (37.1%), cell suspension (32.3%), and control (30.2%) on day 14. This result demonstrates that the design concept of combining stem cells and hydrogels accelerated the wound healing process of burn wound models. The cell spheroid with hydrogel-treated animal group showed a wound area that decreased significantly faster than all other treatment methods on day 10. This indicates that the cell spheroid with hydrogel has a higher efficacy when accelerating wound contraction.

**Figure 3:**
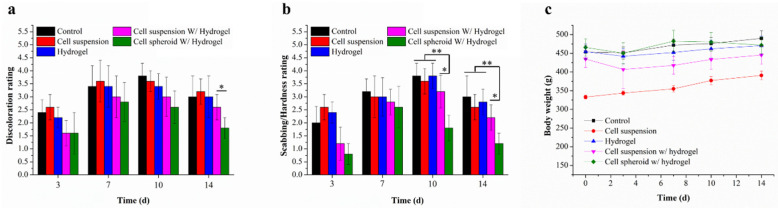
Quantification of wound healing index: (**a**) discoloration and (**b**) scabbing/hardness as a function of wound repair efficacy, assessed by visual observation of wound site photos (n = 4). Samples were analyzed with one-way ANOVA, * *p* < 0.05 and ** *p* < 0.01. Error bars represent mean ± s.d. (**c**) Body weight of the burn wound healing model treated with different formulations.

A wound-healing index was established attempting to quantify efficacy compared to the five treatment groups. The extent of brown discoloration and scabbing/hardness is represented by a score of 0 to 4, and the results are shown in Figure 3. Detail for the wound-healing index are presented in Table 1. After the formation of wounds, all burn wounds gradually turned dark brown, formed scabs, and then returned to a healthy pink shade as the wound healing process began. During the wound healing process, all treatment groups recorded similar discoloration scores, with increasing overall average discoloration score and scabbing/hardness over the first 7 days. After partial thickness burn wound formation, the wound size increased slightly over the first three days and formed eschars compared to the initial wound area. The wound contraction trends are consistent with the formation of scab on the first three days of healing followed by increasing epithelialization and necrotic tissue. In the remaining studies, the wound area started to contract gradually in all treatment groups [38]. Nevertheless, on day 10 and day 14, compared to both the hydrogel and the cell suspension with hydrogel groups, the wound areas treated by the cell spheroid with hydrogel group showed lower discoloration ratings and roughness scores and wound size was also smaller on day 10. The lower discoloration and scabbing of the cell spheroid with hydrogel group is attributed to the reduced extent of scab development. As a result, tissues can regenerate faster.

### 3.3. Body Weight

In order to evaluate the biocompatibility of gelatin hydrogels, body weight was observed during the experiment. The bodyweight variation was not significantly decreased among all groups of rats, the data were shown in Figure 3c. In the initial stage and the final stage of the experiments, the primary evidence shows no serious pathological abnormalities or rapid bodyweight loss during burn wound healing experiments.

### 3.4. Histological Evaluation of Wound Healing

**Figure 4:**
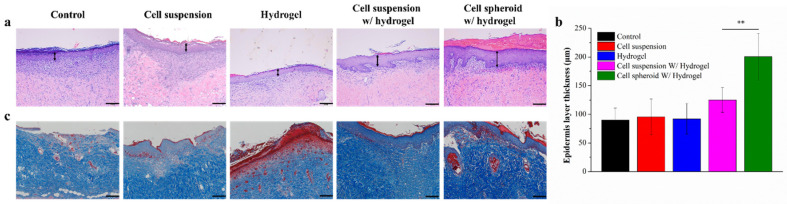
(**a**) Hematoxylin and Eosin (H&E)-stained epidermises from the leading wound edge at 14 days, scale bar: 200 μm; (**b**) bar graph showing epidermal thickness at 14 days, n = 4, with samples analyzed with one-way ANOVA, ** *p* < 0.01, and error bars representing mean ± s.d.; and (**c**) representative photographs of the collagen marker (Masson’s trichrome) for wounds treated with different formulations: collagen was stained in a blue color. Scale bar: 200 μm.

The wound healing ability and local toxicity of the materials implanted in the wound site can be confirmed with histological findings, analysis, and inspection, as shown in Figure 4a and Figure S3. Usually, re-epithelialization and granulation tissue regeneration are important characteristics of skin repair. Appropriate cell migration and optimal cellular infiltration within the wound site can accelerate the wound healing process [39]. After the different wound dressing treatments, the wound site showed various extents of re-epithelialization, regeneration, and repair. The edges of wounds with different treatments revealed different epithelium reformed and maturity levels [40]. To determine whether the hASCs can promote epidermal layer regeneration (Figure 4b), the epidermal layer width was calculated by ImageJ. The control, cell suspension, and hydrogel showed no difference in epidermal layer thickness. At the leading wound edge, cell spheroids with hydrogel treated wounds were associated with significantly thicker epidermises compared with cell suspension with hydrogel. These results suggest that, in addition to accelerated wound healing, the cell spheroid with hydrogel treatment is associated with epidermal thickening and can improve wound architecture.

### 3.5. Masson’s Trichrome Staining

The natural wound healing process involves many cell phenotypes; these cell phenotypes are responsible for rebuilding the tissue structure, which is necessary for cell reassembly [41]. Among these cell types, fibroblasts are highly considered essential matrix remodeling cells which can promote collagen deposition and serve as a temporary matrix for cell growth and tissue regeneration [42]. Collagen deposition was observed by Masson’s trichrome staining in wounds treated with different groups (Figure 4c). Masson’s trichrome staining at the wound site shows that the control group has the least collagen compared to the cell spheroid with hydrogel and the cell suspension with hydrogel treatments. Thus, we proved that hASCs increased collagen synthesis and deposition compared to control group. Moreover, cell spheroid with hydrogel further elevated the rate of collagen synthesis and deposition in comparison to cell suspension with hydrogel. This analysis confirmed that a cell spheroid can induce more collagen synthesis and deposition at the wound sites than the other treatments.

### 3.6. CD31 Immunochemistry Staining

**Figure 5:**
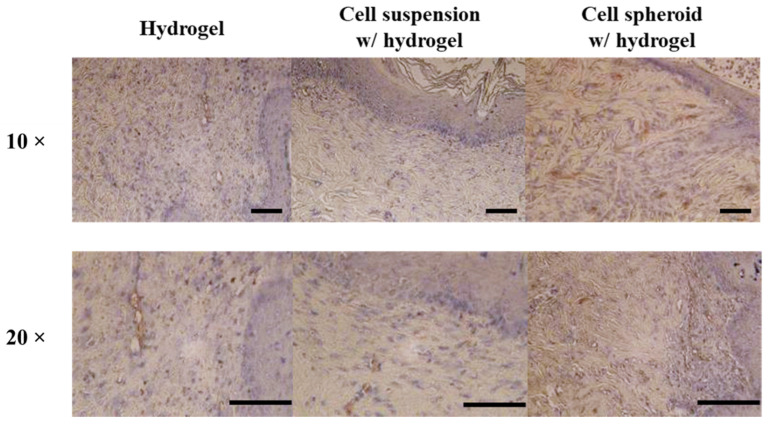
Representative photographs of the angiogenesis state in the wound area and immunohistochemistry staining (brown: CD31) at the wound healing region 14 days after treatment. Scale bar: 200 µm.

In addition to collagen formation, angiogenesis is an indicator that reflects skin regeneration efficacy. The CD31 antibody stained the endothelial cells in a brown color to evaluate the extent of angiogenesis after the wound healing process (Figure 5) [43]. It was found that the microvessels were increased on day 14 in the cell spheroid with hydrogel group relative to other groups. The angiogenesis signal showed a little discrepancy in both the cell suspension with hydrogel and the gelatin hydrogel group. Overall, these results indicate that hASCs can promote wound healing and can trigger neovascularization through their ability to release growth factors such as platelet-derived growth factor (PDGF), vascular endothelial growth factor (VEGF), and basic fibroblast growth factor (bFGF) [44]. Notably, these growth factors are related to angiogenesis in wound healing. The result indicated that hASCs can promote endothelial cells proliferation and can lead to microvessel formation. Since stem cell spheroids increase the function of cellular interactions with the extracellular matrix compared to 2D cultured cells, cell spheroids have a positive effect on the secretion of growth factors [45]. Therefore, compared to the cell suspension with hydrogel group, a cell spheroid with hydrogel can release more growth factors, resulting in a cell spheroid that can promote the formation of microvessels. The observed effects may be mediated through a combination of increased fibroblast proliferation, augmented growth factor production, and enhanced cellular recruitment by promoting re-epithelialization and cell infiltration.

## 4. Conclusions

In this study, an enzyme-crosslinked gelatin hydrogel containing the cell spheroid was successfully prepared and the cell spheroid grew well in the hydrogel. In vivo burn wound healing experiments show that the cell spheroid with hydrogel has a higher efficacy in accelerating wound contraction. Moreover, using the wound healing index (discoloration and scabbing/hardness), we can find that the lower discoloration and scabbing of the cell spheroid with hydrogel group is attributed to the reduced extent of scab development. Therefore, tissues can regenerate faster. Besides, H&E staining images showed that the cell spheroid with hydrogel-treated wounds were associated with significantly thicker epidermises and can improve wound architecture. Spheroids exhibit improved biological properties when compared to cells in suspension or in the hydrogel without direct cell-cell interaction. The spheroids have advantages including enhanced cell viability, stable morphology, increased proliferative activity, and physiological metabolic function [46,47]. We had also demonstrated that, in the in vitro experiments, the cell spheroids maintained higher stemness when undifferentiated but better differentiation capacities when subjected to differentiation conditions [48]. In addition, cell spheroids also carried rich extracellular matrix materials and growth factors, which may lead to better paracrine effects when compared with single cell systems. Masson’s trichrome staining proved that hASCs increased collagen synthesis and deposition compared to the control group. CD31 immunochemistry staining confirms the angiogenesis effect in which the cell spheroid with hydrogel can release more growth factors due to the spheroid having increased cell-cell/cell-ECM interactions compared to 2D cultured cells, leading to increased secretion of growth factors. These results show the feasibility of using the cell spheroid with hydrogel system to facilitate wound repair in the near future.

## Figures and Tables

**Table 1:** Wound healing index of brown discoloration and scabbing/hardness.

Wound Healing Index	Brown Discoloration	Scabbing/Hardness
0	no discoloration	normal skin
1	slight tan color	slight roughness and hardening
		edges not raised
2	light brown in color	moderate roughness and hardening
		slightly raised edges
3	moderate brown in color	hard rough scab and hardening
		moderately raised edges
4	maximum discoloration	hard rough scab

## Supplementary Materials

The following are available online at https://www.mdpi.com/2073-4360/12/12/2997/s1, Figure S1: 2D fluorescence microscope images of the live/dead assay of the cells and cell spheroids proliferated in the gelatin/mTG hydrogel after culture for 7 days (green: live and red: dead) scale bar = 200 mm, Figure S2: Phase contrast microscopy images of the cells and cell spheroids proliferated in the gelatin/mTG hydrogel after culture for 3 and 7 days; scale bar = 200 mm, Figure S3: Histological examination of burn wounds of different treatment groups at post-burn day 14. Abbreviations: ep, epidermis; de, dermis; hf, hair follicle. Scale bar: 200 μm.
