# Supplementary material for: Enzyme-Crosslinked Gelatin Hydrogel with Adipose-Derived Stem Cell Spheroid Facilitating Wound Repair in the Murine Burn Model
Source: Polymers (Basel). 2020 Dec 16;12(12):2997. doi: 10.3390/polym12122997 (PMC7765510; doi:10.3390/polym12122997)
Supplement: Supplementary file 1 [file polymers-12-02997-s001.pdf]

polymers

MDPI

Article

Enzyme-Crosslinked Gelatin Hydrogel with Adipose-Derived Stem Cell Spheroid Facilitating Wound Repair in the Murine Burn Model

Ting-Yu Lu [1], Kai-Fu Yu [1], Shuo-Hsiu Kuo [1], Nai-Chen Cheng [2,*], Er-Yuan Chuang [3,4,*] and Jiashing Yu [1,*]

[1] Department of Chemical Engineering, College of Engineering, National Taiwan University, Taipei 10617, Taiwan; bbb32037@gmail.com (T.-Y.L.); b04504122@gmail.com (K.-F.Y.); b05504084@ntu.edu.tw (S.-H.K.)
[2] Department of Surgery, National Taiwan University Hospital, Taipei 10617, Taiwan
[3] Graduate Institute of Biomedical Materials and Tissue Engineering, International Ph.D. Program in Biomedical Engineering, Taipei Medical University, Taipei 11031, Taiwan
[4] Cell Physiology and Molecular Image Research Center, Wan Fang Hospital, Taipei Medical University, Taipei 11031, Taiwan
* Correspondence: naichenc@gmail.com (N.-C.C.); eychuang@tmu.edu.com (E.-Y.C.); jiayu@ntu.edu.tw (J.Y.)

Received: 11 November 2020; Accepted: 14 December 2020; Published: date

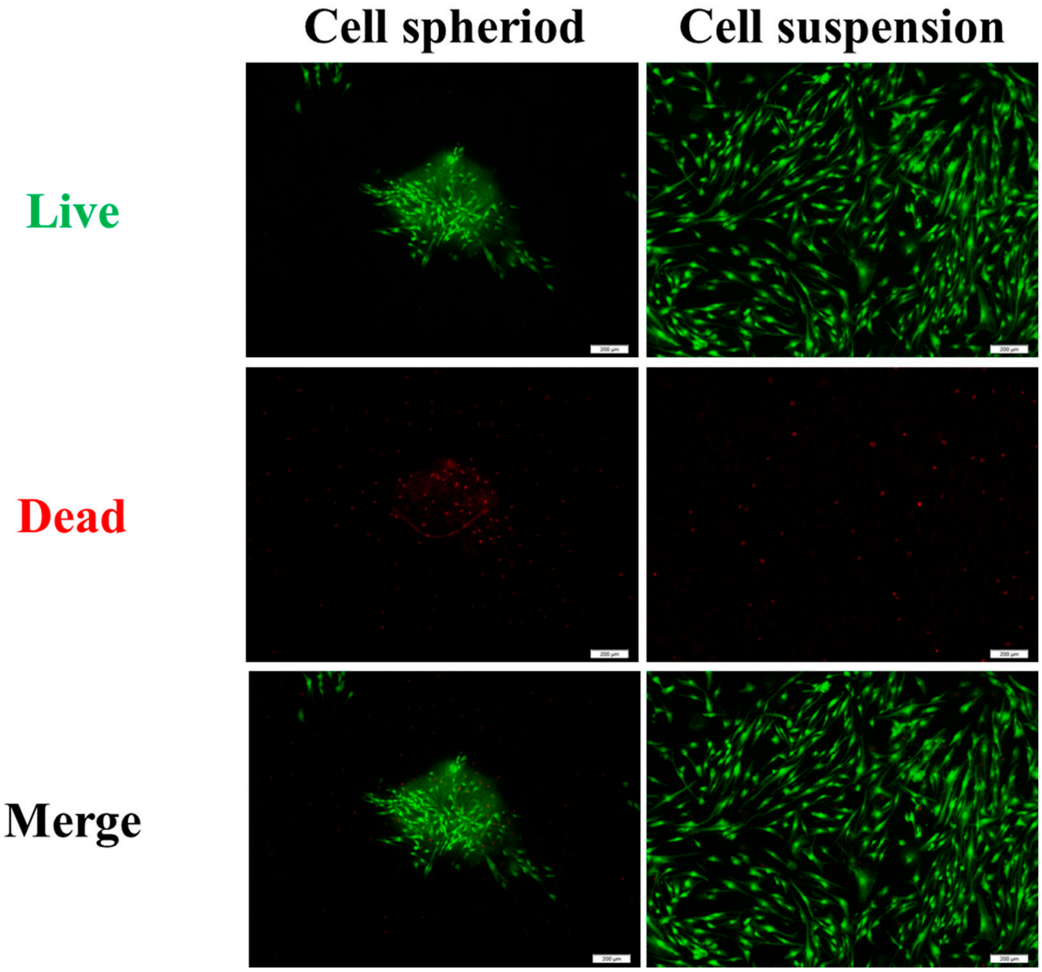

Figure S1. 2D fluorescence microscope images of the live/dead assay of the cells and cell spheroids proliferated in the gelatin/mTG hydrogel after culture for 7 days (green: live and red: dead) scale bar = 200 mm.

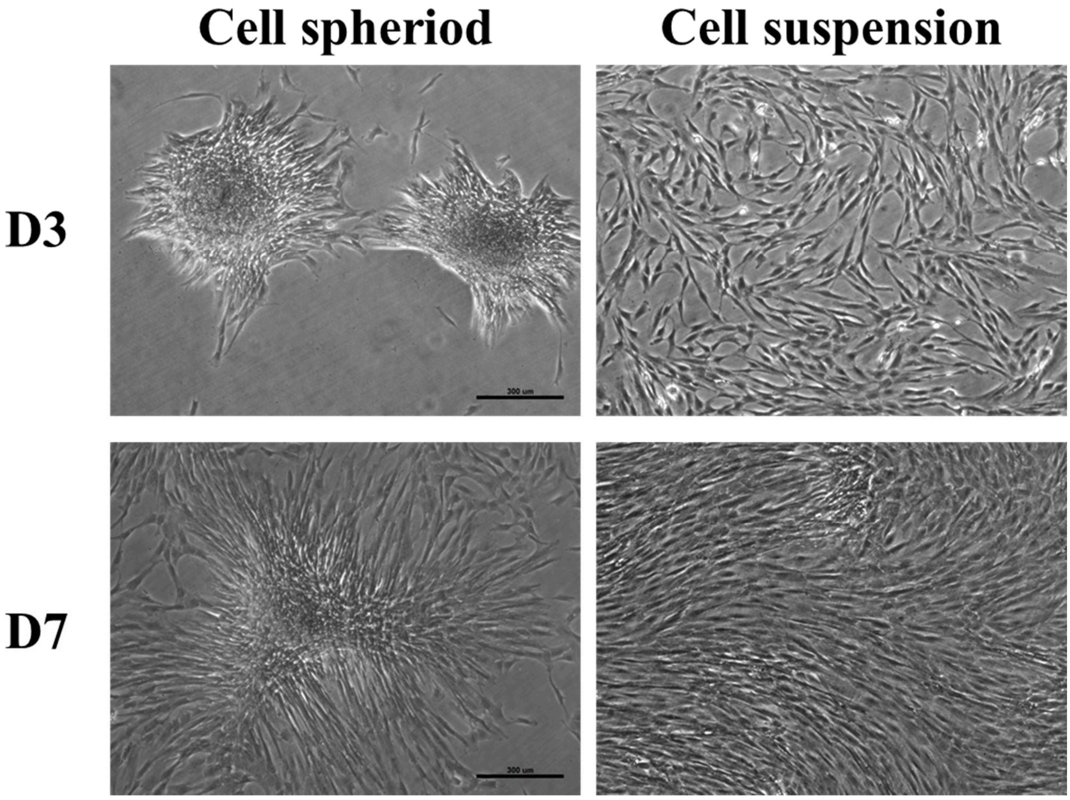

Figure S2. Phase contrast microscopy images of the cells and cell spheroids proliferated in the gelatin/mTG hydrogel after culture for 3 and 7 days; scale bar = 200 mm.

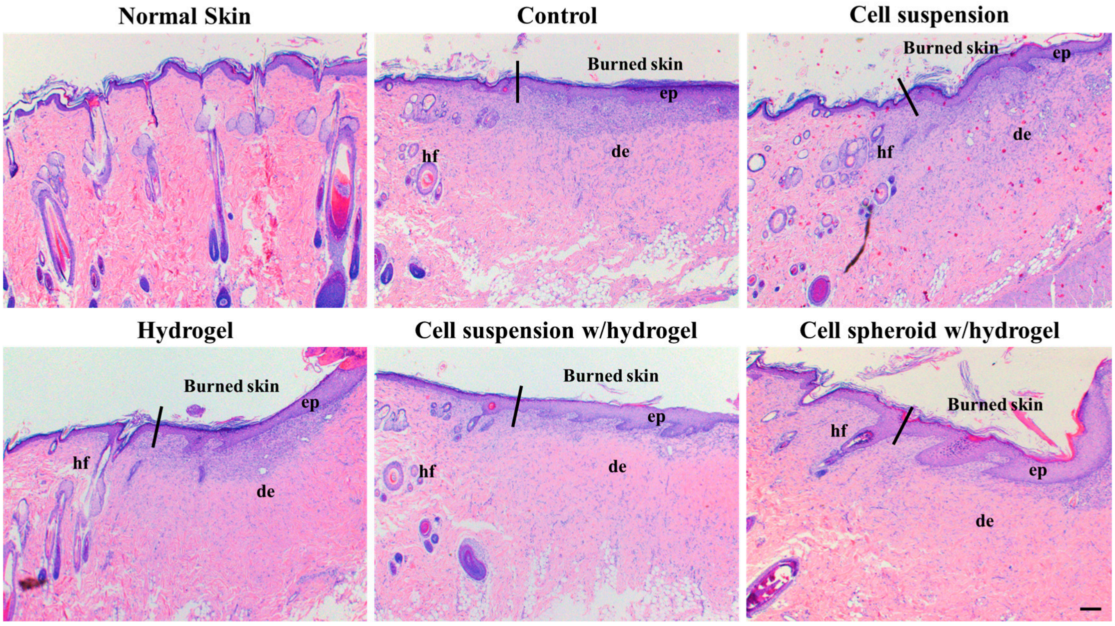

Figure S3. Histological examination of burn wounds of different treatment groups at post-burn day 14. Abbreviations: ep, epidermis; de, dermis; hf, hair follicle. Scale bar: 200 μm.

Publisher's Note: MDPI stays neutral with regard to jurisdictional claims in published maps and institutional affiliations.

© 2020 by the authors. Submitted for possible open access publication under the terms and conditions of the Creative Commons Attribution (CC BY) license (http://creativecommons.org/licenses/by/4.0/).
